# Validation of the German Version of the Movement Disorder Society Non‐Motor Scale (MDS‐NMS)

**DOI:** 10.1002/mdc3.70486

**Published:** 2025-12-22

**Authors:** Jonas Bendig, Anika Frank, Adrianna Lipska‐Dieck, Kristof Wunderlich, David Geißler‐Lösch, Isabel Wurster, Roswitha Kemmner, Kathrin Brockmann, Sheng Luo, Christopher G. Goetz, Glenn T. Stebbins, Pablo Martinez‐Martin, Tiago A. Mestre, Alvaro Sanchez‐Ferro, Monica M. Kurtis, Michelle H.S. Tosin, Roberta Balestrino, Chi‐Ying R. Lin, Carmen Gasca‐Salas, Heinz Reichmann, Bjoern H. Falkenburger

**Affiliations:** ^1^ Department of Neurology, Faculty of Medicine and University Hospital Carl Gustav Carus Technische Universität Dresden Dresden Germany; ^2^ German Center for Neurodegenerative Diseases (DZNE) Dresden Germany; ^3^ Center of Neurology, Department of Neurodegeneration and Hertie‐Institute for Clinical Brain Research University of Tuebingen Tuebingen Germany; ^4^ German Center for Neurodegenerative Diseases University of Tuebingen Tuebingen Germany; ^5^ Department of Biostatistics & Bioinformatics Duke University Durham NC USA; ^6^ Department of Neurological Sciences Rush University Medical Center Chicago IL USA; ^7^ Center for Networked Biomedical Research, Neurodegenerative Diseases (CIBERNED) Carlos III Institute of Health Madrid Spain; ^8^ Parkinson's Disease and Movement Disorders Center, Division of Neurology, Department of Medicine The Ottawa Hospital Research Institute, University of Ottawa Brain and Mind Institute Ottawa Ontario Canada; ^9^ HM CINAC, Hospital Universitario HM Puerta del Sur, Universidad CEU‐San Pablo Madrid Spain; ^10^ Movement Disorders Unit, Neurology Department Hospital Universitario 12 de Octubre Madrid Spain; ^11^ Neurology Department Movement Disorders Unit, Hospital Ruber Internacional Madrid Spain; ^12^ Vita‐Salute San Raffaele University Milan Italy; ^13^ Neuroimaging Research Unit, Division of Neuroscience IRCCS San Raffaele Scientific Institute Milan Italy; ^14^ Neurorehabilitation Unit, IRCCS San Raffaele Scientific Institute Milan Italy; ^15^ Alzheimer's Disease and Memory Disorders Center, Department of Neurology Baylor College of Medicine Houston TX USA; ^16^ Parkinson's Disease Center and Movement Disorders Clinic, Department of Neurology Baylor College of Medicine Houston TX USA

**Keywords:** MDS‐NMS, non‐motor symptoms, Parkinson's disease, validation

Parkinson's disease (PD) is increasingly recognized for its complex spectrum of non‐motor symptoms (NMS). Given their impact, accurate assessment of NMS are essential for optimal PD care. To this end, the initial Non‐Motor Symptom Scale (NMSS)^1^ was revised into the The International Parkinson and Movement Disorder Society—Non‐Motor Rating Scale (MDS‐NMS).^2^, ^3^ Here, we translated the MDS‐NS into German and validate this German version in adherence to the methodology of the official MDS clinical outcome assessment program.

The validation cohort consisted of 300 patients with PD from two sites in Germany (Dresden: *n* = 209; Tübingen: *n* = 91). Methods and demographic characteristics of the study participants are listed in the supplement (Table S1). Cognitive Pretesting was performed with ten patients and three movement disorder specialists. Expert feedback resulted in minor changes in wording to improve clarity of the translation. Most patients found the scale relevant, comprehensive and easy to understand (nine out of ten for each category). Four out of ten patients found questions related to the sexual domain embarrassing, while five out of ten patients found some questions (related to sexual domain, mood or depression) from the scale difficult to answer. Overall, no major problems were identified and the final translation with minor language changes was approved as official working document for the validation phase.

Our study cohort consisted of patients with mainly mild to moderate PD, but nevertheless, we found a high prevalence of NMS as previously reported by others.^4^ Symptoms in the sleep and wakefulness subscale showed the highest prevalence (95.0%) and symptoms on the psychosis subscale displayed the lowest prevalence (30.3%). At least one non‐motor symptom was present in every patient of our cohort (Table S2).

Table 1 displays the confirmatory factor analysis results for each of the 10 subscales of the German MDS‐NMS, which satisfied our pre‐specified criterion of CFI ≥0.90 in comparison with the English‐language factor structure. The exploratory factor analysis results revealed a single factor for all domains of the MDS‐NMS and the Non‐Motor Fluctuations (NMF) subscale (based on scree plots, Fig. S1). Factor loadings for individual items can be found in Table S3.

**TABLE 1:** German Confirmatory Factor Analysis (CFA) results for the 14 subscales (13 domains of the MDS‐NMS plus the NMF subscale)

Item	CMIN	DF	RMSEA	CFI
A. Depression	5.68	5	0.02	1.00
B. Anxiety	0.43	2	0	1.00
C. Apathy	—	—	—	—
D. Psychosis	1.52	2	0	1.00
E. Impulse Control and Related Disorders	0.43	2	0	1.00
F. Cognition	9.36	9	0.01	1.00
G. Orthostatic Hypotension	—	—	—	—
H. Urinary	—	—	—	—
I. Sexual	—	—	—	—
J. Gastrointestinal	0.9	2	0	1.00
K. Sleep and Wakefulness	6.61	9	0	1.00
L. Pain	0.8	2	0	1.00
M. Other	1.05	5	0	1.00
NMF (Fluctuations)	21.1	20	0.01	1.00

*Note*: Note that the CFA could not be performed for the domains apathy, orthostatic hypotension, urinary and sexual as they have three or fewer items and therefore do not possess the necessary degrees of freedom.

Abbreviations: CFI, Comparative Fit Index; CMIN, χ^2^; DF, degrees of freedom; NMF, Non‐Motor Fluctuation; RMSEA, Root Mean Square Error of Approximation.

Limitations of our study include a possible selection bias of specialized movement disorder centers in which participants were recruited and the reliance on only two recruitment sites in Germany. Finally, the cross‐sectional design of our study did not allow the assessment of metrics like test–retest reliability, which should be investigated in longitudinal follow‐up studies.

Based on the results of this validation study, the German version of the MDS‐NMS met the criteria to be considered an MDS‐approved Official Translation and provides a successful cross‐cultural adaptation of the MDS‐NMS.

## Author Roles

(1) Research Project: A. Conception, B. Organization, C. Execution; (2) Statistical Analysis: A. Design, B. Execution, C. Review and Critique; (3) Manuscript Preparation: A. Writing of the First Draft, B. Review and Critique; (4) Responsibility: A. Integrity of the Data, B. Accuracy of the Data Analysis.

J.B.: 1A, 1B, 1C, 2A, 2B, 3A, 4A, 4B

A.F.: 1A, 1B, 1C, 2A, 2B, 3A, 4A, 4B

A.L.D.: 1B, 1C, 3B

K.W.: 1B, 1C, 3B

D.G.L.: 1B, 1C, 3B

I.W.: 1B, 1C, 3B

R.K.: 1B, 1C, 3B

K.B.: 1B, 1C, 3B

S.L.: 1A, 1B, 2A, 2C, 3B, 4A, 4B

C.G.G.: 1A, 1B, 1C, 3B

G.T.S.: 1A, 1B, 1C, 3B

P.M.M.: 1A, 1B, 1C, 3B

T.A.M.: 1A, 1B, 1C, 3B

A.S.F.: 1A, 1B, 1C, 3B

M.M.K.: 1A, 1B, 1C, 3B

M.H.S.T.: 1A, 1B, 1C, 3B

R.B.: 1A, 1B, 1C, 3B

C.Y.R.L.: 1A, 1B, 1C, 3B

C.G.S.: 1A, 1B, 1C, 3B

H.R.: 1A, 1B, 3B

B.H.F.: 1A, 1B, 2A, 2C, 3A, 3B, 4A, 4B

## Disclosures

**Ethical Compliance Statement:** The Ethics Committee of Dresden and Tübingen approved the study. Written informed consent was obtained from all participants by a study investigator. We confirm that we have read the Journal's position on issues involved in ethical publication and affirm that this work is consistent with those guidelines.

**Financial Disclosures and Conflicts of Interest:** This work was supported by a grant from the International Parkinson and Movement Disorder Society (MDS). The authors declare that there are no conflicts of interest relevant to this work.

**Financial Disclosures and Conflicts of Interest:** JB received a fellowship from the German Research foundation as well as the Joachim Herz Foundation and a salary from Columbia University. AF received a fellowship from the German Research foundation and a salary from Columbia University. KB performed Consulting work with honoraria for F. Hoffmann‐La Roche Ltd., Vanqua Bio, and the Michael J. Fox Foundation for Parkinson's Research. She received grant funding from the Michael J. Fox Foundation for Parkinson's Research (“LRRK2 Kinase Activity,” “Influence of Inflammatory Profiles on PD Phenotype and Progression,” “Prevent Dementia in GBA‐associated PD”), from the University of Tübingen (“Endophenotyping of GBA‐PD”), from the German Society for Parkinson DPG, from the Health Forum Baden Wuerttemberg (“Predictive Diagnostic of immune‐associated diseases for personalized medicine”), from the Else Kröner Fresenius Stiftung (“ClinBrain”), and from the German Research Foundation DFG (“CORO‐TREND”). She serves on the advisory board for F. Hoffmann‐La Roche Ltd. She received speaker honoraria from Abbvie, Lundbeck, UCB and Zambon. SL received consulting or advisory board membership with honoraria from the National Institutes of Health, IQVIA, BIAL Biotech, Parkinson Study Group, Huntington Study Group, International Parkinson and Movement Disorder Society; grants/research from the National Institutes of Health, CHDI Foundation/Management, Inc., International Parkinson and Movement Disorder Society, Parkinson's Foundation, and Michael J. Fox Foundation; and a salary from Duke University. CGG received honoraria for consulting or advisory board membership with Theranova. Research conducted by Dr. Goetz at Rush University Medical Center was supported by funding awarded to the center from the National Institutes of Health (NIH), UO1 funding from the National Institute of Diabetes and Digestive and Kidney Diseases (NIDDK), grants from the Department of Defense, and the Michael J. Fox Foundation. Additionally, Dr. Goetz received honoraria as faculty and program management stipends from the International Parkinson and Movement Disorder Society, and received royalties from Elsevier Publishers, Wolters Kluwer, Oxford University Press, and Springer Science. Salary compensation was received from Rush University Medical Center. ASF received grants or contracts from ERA‐NET Horizon 2020 program JPCOFUND2 (reference number HESOCARE‐329‐073), MDS (eDiary project), and Instituto de Salud Carlos III (reference number P122/01177); consulting fees from AbbVie, Boston Scientific, Esteve, Orion Pharma, Prim; and Roche and payment or honoraria for lectures, presentations, speakers bureaus, manuscript writing, or educational events from AbbVie, Bayer, Esteve, MDS Society, EAN, Novartis, Monitor, Organon, Roche, SEN, Stada, Teva, and Zambon. He received a salary from Leuko Labs and Hospital 12 de Octubre and royalties from patents #: 12216112, 20250013841, 12131217, 20240277243, 11963750, 20240085399, 20240085399, 20230032932, 11445980, 20220254024, 11244452, 20210374963, 10959676, 20210038159, 20200060622, 10506979, 20190139221, 20180235548, 9984277, D1044020, 9867573, 20160148038, 20150272504. He owned stock from Leuko Labs. MMK served on the advisory board and performed consulting work with honoraria for Bial. She received honoraria from Eisai, Bial, Esteve and the Spanish Neurological Society. She received grant funding from EU Horizon 2023–2027 and Quironsalud 2023–2024. Ger salary was provided by Neurología Aplicada SLP. CGS performed consulting work with honoraria for Instituto de Salud Carlos III and received grant funding from the Community of Madrid Grant. She received Funding to attend a scientific meeting from Esteve. MHST received a salary from Rush University and is received honoraria for participation in a working group supported by the International Parkinson and Movement Disorder Society. RB received a travel grant from Lusofarmaco. BHF has received advisory and consultancy honoraria from AbbVie, Aleva, Amylyx, Bial, Desitin, Ever Pharma, Stadapharm, Zambon and is an investigator on studies funded by AbbVie, Bial, Biogen, Centogene, CHDI, Denali, DFG, G‐BA, Lundbeck, MDS, MJFF, PD Neurotechnology, Sanofi, Takeda, Teva, UCB. He received a salary from The University Hospital Carl Gustav Carus Dresden. The remaining authors have no additional disclosures to report.

## Supporting information


**Data S1** Supporting Information.
**Supplementary Table 1** Demographic data of the German cohort.
**Supplementary Table 2** Prevalence and scores for non‐motor symptoms.
**Supplementary Table 3** Exploratory factor structure for subscales of the German MDS‐NMS.
**Supplementary Figure 1** Scree plots of Item A‐M and NMF of MDS‐NMS.

## Data Availability

The data that support the findings of this study are available from the corresponding author upon reasonable request.
