# Supplementary material for: Validation of the German Version of the Movement Disorder Society Non‐Motor Scale (MDS‐NMS)
Source: Mov Disord Clin Pract. 2025 Dec 22;13(5):1346–8. doi: 10.1002/mdc3.70486 (PMC13172744; doi:10.1002/mdc3.70486)
Supplement: Supplementary file 1 — Data S1 Supporting Information. Supplementary Table 1 Demographic data of the German cohort. Supplementary Table 2 Prevalence and scores for non‐motor symptoms. Supplementary Table 3 Exploratory factor structure for subscales of the German MDS‐NMS. Supplementary Figure 1 Scree plots of Item A‐M and NMF of MDS‐NMS. [file MDC3-13-1346-s001.docx]

**Supplement to:**

Validation of the German Version of the Movement Disorder Society Non-Motor Scale (MDS-NMS)

Jonas Bendig^1^, Anika Frank^1,2^, Adrianna Lipska-Dieck^1^, Kristof Wunderlich^1^, David Geißler-Lösch^1^, Isabel Wurster^3,4^, Roswitha Kemmner^3,4^, Kathrin Brockmann^3,4^, Sheng Luo^5^, Christopher G. Goetz^6^, Glenn T. Stebbins^6^, Pablo Martinez-Martin^7^, Tiago A. Mestre^8^, Alvaro Sanchez-Ferro^9,10^, Monica M. Kurtis^11^, Michelle HS Tosin^6^, Roberta Balestrino^12,13,14^, Chi-Ying R Lin^15,16^, Carmen Gasca-Salas^7,9^, Heinz Reichmann^1^, Bjoern H. Falkenburger^1,2^

^1^Department of Neurology, Faculty of Medicine and University Hospital Carl Gustav Carus, Technische Universität Dresden, Dresden, Germany.

^2^German Center for Neurodegenerative Diseases (DZNE), Dresden, Germany.

^3^Center of Neurology, Department of Neurodegeneration and Hertie-Institute for Clinical Brain Research, University of Tuebingen, Tuebingen, Germany. kathrin.brockmann@uni-tuebingen.de.

^4^German Center for Neurodegenerative Diseases, University of Tuebingen, Tuebingen, Germany.

^5^Department of Biostatistics & Bioinformatics Duke University Durham North Carolina USA.

^6^Department of Neurological Sciences, Rush University Medical Center, Chicago, IL, USA.

^7^Center for Networked Biomedical Research, Neurodegenerative Diseases (CIBERNED), Carlos III Institute of Health, Madrid, Spain

^8^Parkinson's Disease and Movement Disorders Center, Division of Neurology, Department of Medicine, The Ottawa Hospital Research Institute, University of Ottawa Brain and Mind Institute, Ottawa, Canada

^9^HM CINAC, Hospital Universitario HM Puerta del Sur, Universidad CEU-San Pablo, Madrid, Spain.

^10^Movement Disorders Unit, Neurology Department, Hospital Universitario 12 de Octubre, Madrid, Spain.

^11^Neurology Department, Movement Disorders Unit, Hospital Ruber Internacional, Madrid, Spain.

^12^Vita-Salute San Raffaele University, Milan, Italy.

^13^Neuroimaging Research Unit, Division of Neuroscience, IRCCS San Raffaele Scientific Institute, Milan, Italy.

^14^Neurorehabilitation Unit, IRCCS San Raffaele Scientific Institute, Milan, Italy.

^15^Alzheimer's Disease and Memory Disorders Center, Department of Neurology, Baylor College of Medicine, Houston, 77030 TX, USA.

^16^Parkinson's Disease Center and Movement Disorders Clinic, Department of Neurology, Baylor College of Medicine, Houston, 77030 TX, USA.

# **Supplementary Methods**

**Study Design and Patient Cohort**

This cross-sectional observational study was conducted at two movement disorder centers in Germany (Dresden and Tuebingen). German-speaking patients with idiopathic PD were invited to participate as defined by the MDS diagnostic criteria. [9] Patients of all ages, genders, and disease stages were included. Each site obtained approval of the responsible ethics committee (Dresden: BO-EK-149032021; Tübingen: 26/2007BO1, 404/2020BO1, 199/2011BO1, 702/2013/BO1). Patients provided signed consent before participating in this study. The sample size was determined by the requirement of five subjects per questionnaire item, with the MDS-NMS containing 60 items, a minimum sample size of 300 participants was necessary.

**Development of the German version of the MDS-NMS**

The German translation followed the phases (translation, back‐translation, comparison with the original and amendments, and cognitive pretesting) of the prescribed protocol established by the MDS COA program for official translations of the MDS‐NMS.

First, a forward translation was developed by a bilingual movement disorder clinician of the translation team. This first version was reviewed by three additional movement disorder specialists, and a draft translated scale was developed based on feedback. Second, a back‐translation was obtained from an independent English-speaking movement disorder specialist and reviewed as well as corrected by a second team consisting of two bilingual movement disorder specialists and a bilingual psychologist with extensive experience in PD. The back-translation was reviewed by the MDS Translation Steering Committee, and the translation was then approved for Cognitive Pretesting, which is a qualitative approach for assessing instrument completion regarding task difficulty for examiner and respondent, and respondent interest, attention span, discomfort, and comprehension. For Cognitive Pretesting, the provisionally approved translation was administered to 10 patients with PD. This phase identifies potentially culturally sensitive or too-difficult items. No further problems were noted. The MDS (COA program) approved the final translation, and field testing was performed on a large sample of native German speakers.

**Assessments**

Socio‐demographic data and disease duration were obtained from all patients with PD through a short questionnaire. Hoehn and Yahr stage was determined by the clinician at the time of questionnaire administration. In addition, a chart review was conducted for the Dresden cohort (200 patients) to identify the UPDRS III rating from routine clinical visits closest to the scale administration, and values in a timeframe of +/- 3 months from the assessment date were included in the analysis. The MDS-NMS was then administered which consists of 52 questions, grouped into 13 domains of non-motor symptoms: (A) Depression (5 questions), (B) Anxiety (4 questions), (C) Apathy (3 questions), (D) Psychosis (4 questions), (E) Impulse Control and Related Disorders (4 questions), (F) Cognition (6 questions), (G) Orthostatic Hypotension (2 questions), (H) Urinary (3 questions), (I) Sexual (2 questions), (J) Gastrointestinal (4 questions), (K) Sleep and Wakefulness (6 questions), (L) Pain (4 questions), and (M) Other (5 questions: Weight loss without intention, reduced smell, fatigue (physical), fatigue (mental), excessive sweating). All questions were first rated for frequency (0 [never] to 4 [majority of time]) followed by a rating for severity (0 [not present] to 4 [severe]). Frequency and severity ratings were multiplied to generate a total score (0 to 16) for each question. Domain scores were calculated by summing the corresponding questions, and the total score was calculated by summing domain scores (0 to 832 points). The MDS-NMS contains an additional Non‐Motor Fluctuations (NMF) subscale with eight questions covering the following symptoms: Depression, anxiety, thinking/cognitive abilities, bladder symptoms, restlessness, pain, fatigue, and excessive sweating. For each symptom, the rater assesses the typical degree of change from “on” to “off” periods (0 [no change) to 4 [large]). The total score of the NMF subscale (0 to 128 points) is calculated from the sum of the degree of change for the eight symptoms (0 to 32 points) multiplied with the amount of time spent in the “off” state with NMS (1 [rarely] to 4 [majority of time]).

**Data Analysis**

*Factor Analysis*

Primary confirmatory and secondary exploratory factor analyses were performed in R (Version 4.2.0) with packages lavaan and psych, respectively. Factor estimation was performed with the adjusted weighted least squares (WLSMV) approach and the orthogonal CF-VARIMAX rotation.

*Primary Analysis*

For the primary analysis of the German data, we conducted a confirmatory factor analysis (CFA) to determine if the factor structure for the English language MDS-NMS(11) could be confirmed in data collected using the German translation. This was the primary question of interest. The CFA was conducted separately for each of the 14 subscales (13 domains of the MDS-NMS plus the NMF subscale), with the German data constrained to fall into the factors defined in the English language data. We evaluated the CFA results using the Comparative Fit Index (CFI). According to the protocol, to establish a successful translation and to designate that translation as an OFFICIAL MDS translation of the MDS-NMS, we required that the CFI for each Part (I-IV) of the translated MDS-NMS be 0.90 or greater relative to the English language version. We used the mean and variance adjusted weighted least square (WLSMV) estimator to confirm the model's fit.

*Secondary Analysis*

As a secondary analysis, we conducted an exploratory factor analysis for each of the 14 subscales (13 domains of the MDS-NMS plus the NMF subscale) of the German version of the MDS-NMS Parts I-IV to explore the underlying factor structure without the constraint of a pre-specified factor structure. With the chosen factors, an item was kept in each factor when the factor loading for that item was 0.40 or greater. To assist in interpreting the factors, an orthogonal CF-VARIMAX rotation was used, which sets the factors to be uncorrelated.

*Cohort-based analyses of non-motor symptom burden in German patients*

We explored the burden of NMS and fluctuations in our patient population based on the MDS-NMS. We defined the prevalence of NMS or fluctuations with a score of ≥1 in each subdomain/item, respectively. In addition, we investigated if the MDS-NMS captures previously described differences in NMS between men and women with PD in our German-speaking cohort. Tertiary analysis was performed in Python (3.11.5) with packages pandas, numpy, scipy, pingouin, statsmodels, matplotlib, and seaborn. The normality of the data was assessed with a Shapiro Wilk Test and by visual inspection of histograms. As data obtained from the MDS-NMS showed non-normal distributions, we used the Mann-Whitney-U (continuous or ordinal variables) or chi-squared test (categorical variables) for comparisons between groups and Spearman's ρ to assess the correlation between variables.

**Supplementary Table 1.** Demographic data of the German cohort

| Variable |  |
| --- | --- |
| n | 300 |
| Men, N (%) | 193 (64.3) |
| Race, N (%) |  |
| White  Missing | 294 (98.0)  6 (2.0) |
| Age, years |  |
| Mean (SD) | 65.5 (10.5) |
| PD diagnosis, years |  |
| Mean (SD) | 8.55 (6.35) |
| Formal education, years |  |
| Mean (SD) | 15.11 (3.12) |
| Hoehn and Yahr stage, N (%) |  |
| 1 | 33 (11.0) |
| 2 | 171 (57.0) |
| 3 | 74 (24.7) |
| 4 | 12 (4.0) |
| 5 | 0 (0.0) |
| Missing | 10 (3.3) |

Abbreviations: SD, standard deviation; PD, Parkinson’s Disease

**Supplementary Table 2.** Prevalence and scores for non-motor symptoms

|  | **Score** | **Prevalence** |
| --- | --- | --- |
| **Variable** |  |  |
| MDS-NMS, Total | 128.28 ± 89.58 | 100% (300) |
| Depression Subscale | 10.88 ± 14.95 | 74.67% (224) |
| Anxiety Subscale | 10.11 ± 11.18 | 82.33% (247) |
| Apathy Subscale | 5.85 ± 8.29 | 64.33% (193) |
| Psychosis Subscale | 2.16 ± 6.64 | 30.33% (91) |
| Impulse Control and Related Disorders Subscale | 3.17 ± 5.75 | 44.67% (134) |
| Cognition Subscale | 13.86 ± 16.36 | 88.67% (266) |
| Orthostatic Subscale | 5.61 ± 8.17 | 63.33% (190) |
| Urinary Subscale | 12.24 ± 12.87 | 83.67% (251) |
| Sexual Subscale | 5.97 ± 8.26 | 57.33% (172) |
| Gastrointestinal Subscale | 10.03 ± 11.05 | 82.67% (248) |
| Sleep and Wakefulness Subscale | 16.04 ± 13.89 | 95.0% (285) |
| Pain Subscale | 15.98 ± 14.16 | 89.67% (269) |
| Other Subscale | 16.37 ± 14.84 | 90.67% (272) |
| NMF, Total | 2.98 ± 5.33 | 31.33% (94) |

Prevalence is defined as a Score of ≥ 1 on any subscale or total scale. Scores show mean and standard deviation. Abbreviations: MDS-NMS - The International Parkinson and Movement Disorder Society – Non-Motor Rating Scale, NMF = Non-Motor Fluctuation.

**Supplementary Table 3. Exploratory factor structure for subscales of the German MDS-NMS**

| \| **Factor** \| **Symptoms** \| **Loading** \| \| --- \| --- \| --- \| \| **A: Depression** \| \| \| \| **Factor 1** \| *Percent variance* \| *50.7* \| \|  \| 1. Felt sad or depressed? \| 0.72 \| \|  \| 2. Had difficulty experiencing pleasure? \| 0.64 \| \|  \| 3. Felt hopeless? \| 0.78 \| \|  \| 4. Had negative thoughts about yourself? \| 0.66 \| \|  \| 5. Felt that life is not worth living? \| 0.76 \| \| **B: Anxiety** \| \| \| \| **Factor 1** \| *Percent variance* \| *33.8* \| \|  \| 1. Felt worried? \| 0.80 \| \|  \| 2. Felt nervous? \| 0.45 \| \|  \| 3. Had panic or anxiety attacks? \| 0.58 \| \|  \| 4. Been worried about being in public or in social situations? \| 0.42 \| \| **C: Apathy** \| \| \| \| **Factor 1** \| *Percent variance* \| *37.1* \| \|  \| 1. Had a reduced motivation to start day-to-day activities? \| 0.62 \| \|  \| 2. Had a reduced interest in talking to people? \| 0.59 \| \|  \| 3. Had a reduction in experiencing emotions? \| 0.61 \| \| **D: Psychosis** \| \| \| \| **Factor 1** \| *Percent variance* \| *52.8* \| \|  \| 1. Sensed things or people in the margins of your visual field? (passage or presence phenomena) \| 0.96 \| \|  \| 2. Visually misinterpreted an actual object? (illusions) \| 0.68 \| \|  \| 3. Seen, heard, felt, tasted, or smelled things that other people did not? (hallucinations) \| 0.77 \| \| **Not loaded** \| 4. Believed things to be true that others did not? (e.g., delusions of persecution, jealousy, or misidentification) \|  \| \| **E: Impulse Control and Related Disorders** \| \| \| \| **Factor 1** \| *Percent variance* \| *23.9* \| \|  \| 2. Had an increase in other behaviors (e.g., internet use, hobbies, artistic activities, writing, hoarding)? \| 0.90 \| \| **Not loaded** \| 1. Had an increase in gambling, sexual, buying, or eating behaviors? \|  \| \| 3. Repeatedly handled objects without any purpose? (punding) \|  \| \| 4. Routinely taken more anti-parkinsonian medications than prescribed? (dopamine dysregulation syndrome) \|  \| \| **F: Cognition** \| \| \| \| **Factor 1** \| *Percent variance* \| *39.9* \| \|  \| 1. Had difficulty remembering things? \| 0.71 \| \|  \| 2. Had difficulty learning new things? \| 0.76 \| \|  \| 3. Had difficulty keeping focus or paying attention? \| 0.57 \| \|  \| 4. Had difficulty finding words or expressing ideas? \| 0.74 \| \|  \| 5. Had difficulty planning or carrying out complex tasks, not due to motor problems? (executive abilities) \| 0.54 \| \| **Not loaded** \| 6. Had difficulty judging the position of things? (visuospatial abilities) \|  \| \| **G: Orthostatic Hypotension** \| \| \| \| **Factor 1** \| *Percent variance* \| *43.6* \| \|  \| 1. Felt lightheaded or fainted when changing position? \| 0.66 \| \|  \| 2. Had dizziness or weakness upon standing? \| 0.66 \| \| **H: Urinary** \| \| \| \| **Factor 1** \| *Percent variance* \| *48.6* \| \|  \| 1. Had an urgent need to empty bladder? (urinary urgency) \| 0.67 \| \|  \| 2. Had to empty bladder more than every 2 hours? (urinary frequency) \| 0.79 \| \|  \| 3. Had to empty bladder more than twice overnight? (nocturia) \| 0.62 \| \| **I: Sexual** \| \| \| \| **Factor 1** \| *Percent variance* \| *39.9* \| \|  \| 1. Had decreased sexual drive or interest in sex? \| 0.63 \| \|  \| 2. Had difficulty with sexual arousal (e.g., erectile dysfunction or vaginal dryness) or sexual performance not related to motor problems (e.g., not related to Parkinson’s rigidity)? \| 0.63 \| \| **J: Gastrointestinal** \| \| \| \| **Factor 1** \| *Percent variance* \| *19.8* \| \|  \| 1. Had any drooling of saliva? \| 0.53 \| \|  \| 2. Had difficulty swallowing? \| 0.56 \| \| **Not loaded** \| 4. Had constipation? (defined as < 3 bowel movements/week) \|  \| \| 3. Had nausea or felt sick in the stomach? \|  \| \| **K: Sleep and Wakefulness** \| \| \| \| **Factor 1** \| *Percent variance* \| *20.1* \| \|  \| 2. Acted out dreams while asleep, such as shouting, flailing arms, punching, or running movements? (REM sleep behavior) \| 0.56 \| \|  \| 4. Had an irresistible urge to move legs or arms when sitting or lying down, which improved with movement? (restlessness) \| 0.40 \| \|  \| 5. Had any involuntary jerky movements in arms or legs during sleep or while resting? (periodic limb movements) \| 0.62 \| \|  \| 6. Woken at night due to snoring, gasping, or difficulty with breathing? \| 0.41 \| \| **Not loaded** \| 1. Had difficulty falling asleep or staying asleep? (insomnia) \|  \| \| 3. Dozed off or fallen asleep unintentionally during waking hours? (e.g., during conversation, at mealtimes, or while driving, watching television; excessive daytime sleepiness) \|  \| \| **L: Pain** \| \| \| \| **Factor 1** \| *Percent variance* \| *28.7* \| \|  \| 1. Had muscle, joint, or back pain? \| 0.66 \| \|  \| 2. Had a deep or dull aching pain within the body? \| 0.45 \| \|  \| 3. Had pain due to abnormal twisting movements of arms or legs or body, often present in the early morning period? (dystonia) \| 0.60 \| \| **Not loaded** \| 4. Had other types of pain? (e.g., nocturnal pain, orofacial pain) \|  \| \| **M: Other** \| \| \| \| **Factor 1** \| *Percent variance* \| *26.9* \| \|  \| 3. Felt excessively physically tired? (physical fatigue) \| 0.70 \| \|  \| 4. Felt excessively mentally tired? (mental fatigue) \| 0.73 \| \|  \| 5. Had excessive sweating not related to temperature? \| 0.43 \| \| **Not loaded** \| 1. Had an unintentional weight loss? \|  \| \| 2. Had a decrease in sense of smell? (impaired olfaction) \|  \| \|  \| **Non-Motor Fluctuations (NMF)** \|  \| \| **Factor 1** \| *Percent variance* \| *37.1* \| \|  \| 1. Depression (as listed in Subscale A) \| 0.61 \| \|  \| 2. Anxiety (as listed in Subscale B) \| 0.68 \| \|  \| 3. Thinking or cognitive abilities (as listed in Subscale F) \| 0.69 \| \|  \| 4. Bladder symptoms (as listed Subscale H) \| 0.49 \| \|  \| 5. Restlessness (as listed in Subscale K, item 4) \| 0.68 \| \|  \| 6. Pain (as listed in Subscale L) \| 0.58 \| \|  \| 7. Fatigue (as listed in Subscale M, items 3 and 4) \| 0.68 \| \| **Not loaded** \| 8. Excessive sweating (as listed in Subscale M, item 5) \|  \| \| *The number of factors is selected based on scree plots in Figure 1. \| \| \| |
| --- | --- | --- | --- | --- | --- | --- | --- | --- | --- | --- | --- | --- | --- | --- | --- | --- | --- | --- | --- | --- | --- | --- | --- | --- | --- | --- | --- | --- | --- | --- | --- | --- | --- | --- | --- | --- | --- | --- | --- | --- | --- | --- | --- | --- | --- | --- | --- | --- | --- | --- | --- | --- | --- | --- | --- | --- | --- | --- | --- | --- | --- | --- | --- | --- | --- | --- | --- | --- | --- | --- | --- | --- | --- | --- | --- | --- | --- | --- | --- | --- | --- | --- | --- | --- | --- | --- | --- | --- | --- | --- | --- | --- | --- | --- | --- | --- | --- | --- | --- | --- | --- | --- | --- | --- | --- | --- | --- | --- | --- | --- | --- | --- | --- | --- | --- | --- | --- | --- | --- | --- | --- | --- | --- | --- | --- | --- | --- | --- | --- | --- | --- | --- | --- | --- | --- | --- | --- | --- | --- | --- | --- | --- | --- | --- | --- | --- | --- | --- | --- | --- | --- | --- | --- | --- | --- | --- | --- | --- | --- | --- | --- | --- | --- | --- | --- | --- | --- | --- | --- | --- | --- | --- | --- | --- | --- | --- | --- | --- | --- | --- | --- | --- | --- | --- | --- | --- | --- | --- | --- | --- | --- | --- | --- | --- | --- | --- | --- | --- | --- | --- | --- | --- | --- | --- | --- | --- | --- | --- | --- | --- | --- | --- | --- | --- | --- | --- | --- | --- | --- | --- | --- | --- | --- | --- | --- | --- | --- | --- | --- | --- | --- | --- | --- | --- | --- | --- | --- | --- | --- | --- | --- | --- | --- | --- | --- | --- | --- | --- | --- | --- | --- | --- | --- | --- | --- | --- | --- | --- | --- | --- | --- | --- | --- | --- | --- |

**Supplementary Figures**

**
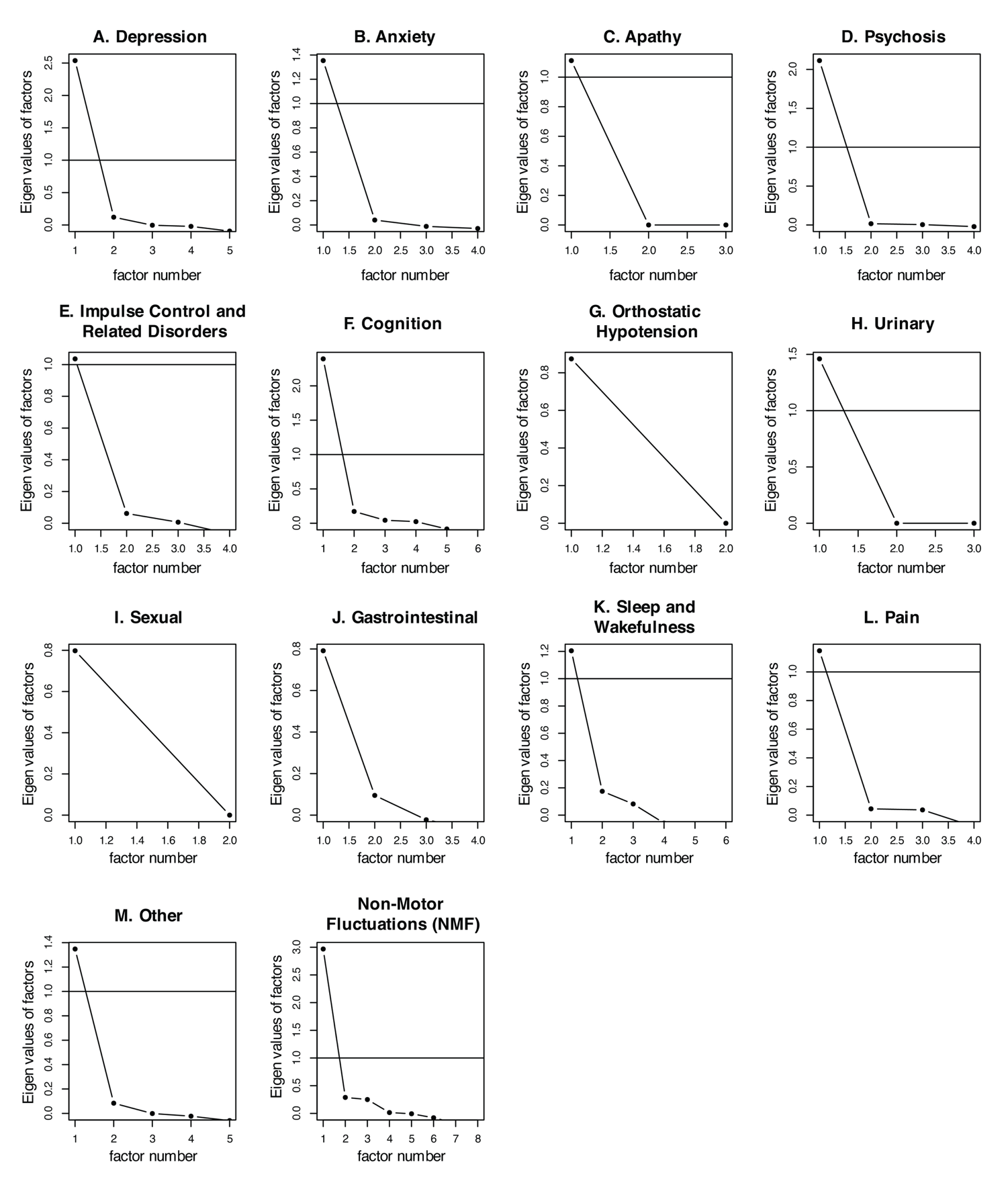
**

**Supplementary Figure 1.** Scree plots of Item A-M and NMF of MDS-NMS
